# Editorial: Global excellence in toxicology: Asia, Australia and New Zealand

**DOI:** 10.3389/ftox.2025.1584009

**Published:** 2025-03-17

**Authors:** Yongning Wu

**Affiliations:** NHC Key Laboratory of Food Safety Risk Assessment, China National Center for Food Safety Risk Assessment, Beijing, China

**Keywords:** food safety, food toxicology, food contaminants regulatory risk assessment, new approach methodologies (NAMs), next generation risk assessment (NGRA)

The rapid pace of industrial development and technological progress has significantly increased environmental pollution and its impact on human health (Huang, 2021; Liew and Guo, 2022). As new pollutants emerge, there is an urgent need for innovative toxicological methods and next-generation risk assessment (NGRA) strategies to address the associated health risks (Schmeisser et al., 2023). The present compilation of articles in *Frontiers in Toxicology* provides a comprehensive overview of the cutting-edge advancements in environmental and health safety in Asia. These studies not only highlight the region’s proactive stance in addressing emerging contaminants but also underscore the integration of novel methodologies in risk assessment and toxicological research. This editorial synthesizes the key insights from these papers and discuss their implications for the future of environmental health and safety in the region and beyond.

The rapid industrialization and urbanization in the Southeast Asia region have led to an alarming increase in the presence of unconventional pollutants, necessitating a paradigm shift in toxicological evaluation. The review article from (Bhagat et al.) offers a sobering account of the escalating environmental threats posed by pharmaceuticals and personal care products (PPCPs), endocrine-disrupting compounds (EDCs), Flame retardants, microplastics, and per-and poly-fluoroalkyl substances (PFAS) in Southeast Asian countries. The tropical climate and diverse agricultural and industrial systems add to the complexity of contamination pathways, emphasizing the urgency for rigorous research and targeted interventions in the region. As stated by the authors, despite facing unique environmental challenges, comprehensive studies on emerging contaminants, in Southeast Asia remain lacking, leaving the regulatory frameworks, policy decisions, and mitigation strategies incomplete. The review also emphasizes the urgency of developing robust toxicological testing methods to mitigate the impacts of these emerging contaminants. The authors call for a comprehensive understanding of the complex interactions among a wide range of chemicals that move through ecosystems, signaling a shift from studying single or grouped chemicals to understanding their collective environmental impact.

To tackle the challenges posed by environmental pollution to human health, NGRA strategies are being developed. The NGRA strategies aim to integrate comprehensive new approach methodologies (NAMs) into the regulatory framework (Cattaneo et al., 2023). NAMs are designed to serve as toxicological alternatives to traditional animal testing-based methods. The article from China National Center for Food Safety Risk Assessment (CFSA) outlines the progress and challenges in developing NAMs for NGRA in China (Yang et al.). As a representative initiative, the “Food Toxicology Program” provides scientific support for food safety risk assessment and management by establishing a systematic toxicological database and exploring alternative methods for hazard assessment. The review underscores the importance of interdisciplinary collaboration and the development of modern toxicology to ensure food safety and environmental sustainability in the region and beyond.

In the application of non-animal alternative methods, the field of cosmetic toxicology safety assessment is relatively advanced. This progress is largely attributed to the implementation of innovative testing methodologies (Hartung, 2009). A study from L’Oréal Research and Innovation group demonstrated how OECD Guidance documents facilitated the transfer of *in vitro* approaches for regulatory acceptance by establishing the EpiSkin™skin irritation test (Liu et al.), which aligns with OECD guidelines on Good *In Vitro* Method Practices (GIVIMPs). The cosmetics safety assessment serves as a model for the broader adoption of alternative testing methods across various sectors, reducing reliance on animal testing while ensuring regulatory compliance and scientific rigor (Nelson et al., 2024). As another alternative method, the human Cell Line Activation Test (h-CLAT) was used to evaluate the immunotoxicity potential of various nanomaterials at the cellular level in THP-1 cells (Nishida et al.). The h-CLAT, an internationally standardized method for assessing the skin sensitization potential of chemicals with guaranteed reliability (OECD, 2024), proves useful in evaluating and comparing the activation potentials of antigen-presenting cells (APCs). In this study, the authors proposed that APCs activation by nanomaterials could serve as a high-throughput method to assess their immunotoxicity. However, constructing a consistent and reliable *in vitro to in vivo extrapolation* (IVIVE) model remains crucial for linking *in vitro* findings to *in vivo* toxicity. In addition, macrophage-like cells derived through the differentiation of THP-1 cells have been highlighted as an important model cell type for evaluating immunometabolic disruption of environmental pollutants, such as phthalates (Schmidt, 2022; Xu et al., 2022). In the future, various human-derived immune cells may offer efficient *in vitro* alternative models for the identification and assessment of immunotoxicity, which is one of the most sensitive targets of environmental pollutants.

In summary, as the landscape of environmental pollutants evolves, so too must our strategies for assessing and managing their risks. The development and implementation of NGRA strategies are essential for safeguarding both environmental and public health in the face of new and emerging pollutants. These articles collectively depict a region at the forefront of innovation in environmental and health safety. They demonstrate a clear trend towards the adoption of alternative testing methodologies that are not only more humane but also scientifically advanced. The development and implementation of NAMs are poised to revolutionize risk assessment and toxicological research in Asia. These advancements are not merely regional achievements, they contribute to the global effort to protect the environment and public health through science-based, innovative, and sustainable approaches.

## References

[B1] Cattaneo I. Astuto M. C. Binaglia M. Devos Y. Dorne J. L. C. M. Fernandez Agudo A. (2023). Implementing new approach methodologies (NAMs) in food safety assessments: strategic objectives and actions taken by the European food safety authority. Trends Food Sci. and Technol. 133: 277–290. 10.1016/j.tifs.2023.02.006

[B2] Hartung T. (2009). Toxicology for the twenty-first century. Nature. 460(7252):208–212. 10.1038/460208a 19587762

[B3] Huang Y. (2021). Technology innovation and sustainability: challenges and research needs. Clean. Techn Environ. Policy 23, 1663–1664. 10.1007/s10098-021-02152-6 34276276 PMC8273565

[B4] Liew Z. Guo P. (2022). Human health effects of chemical mixtures. Science. 375 (6582):720–721. 10.1126/science.abn9080 35175805 PMC9805352

[B5] Nelson C. P. Brown P. Fitzpatrick S. Ford K. A. Howard P. C. MacGill T. (2024). Advancing alternative methods to reduce animal testing. Science. 386(6723):724–726. 10.1126/science.adg6228 39541448

[B6] OECD (2024). Test No. 442E: in vitro skin sensitisation: in vitro skin sensitisation assays addressing the key event on activation of dendritic cells on the Adverse Outcome pathway for skin sensitisation, OECD guidelines for the testing of chemicals, section 4, Paris, France: OECD Publishing, 10.1787/9789264264359-en

[B7] Schmeisser S. Miccoli A. von Bergen M. Berggren E. Braeuning A. Busch W. (2023). New approach methodologies in human regulatory toxicology - not if, but how and when! Environ. Int. 178: 108082, 10.1016/j.envint.2023.108082 37422975 PMC10858683

[B8] Schmidt S. (2022). Targeting the macrophage: immune cells may Be the key to phthalate-induced liver toxicity. Environ. Health Perspect. 130(3): 34003, 10.1289/EHP11026 35319255 PMC8942079

[B9] Xu M. Li Y. Wang X. Zhang Q. Wang L. Zhang X. (2022). Role of Hepatocyte- and macrophage-specific PPARγ in hepatotoxicity induced by Diethylhexyl phthalate in Mice. Environ. Health Perspect. 130(1): 17005, 10.1289/EHP9373 35019730 PMC8754100

